# Systematic Review of the Relationship between Amyloid-β Levels and Measures of Transgenic Mouse Cognitive Deficit in Alzheimer’s Disease

**DOI:** 10.3233/JAD-142208

**Published:** 2015-01-01

**Authors:** Avery M. Foley, Zeena M. Ammar, Robert H. Lee, Cassie S. Mitchell

**Affiliations:** Department of Biomedical Engineering, Georgia Institute of Technology and Emory University, Atlanta, GA, USA

**Keywords:** Amyloid-β, cognitive deficit, memory, Morris water maze, mouse model, novel object recognition, Tg2576

## Abstract

Amyloid-β (Aβ) is believed to directly affect memory and learning in Alzheimer’s disease (AD). It is widely suggested that there is a relationship between Aβ_40_ and Aβ_42_ levels and cognitive performance. In order to explore the validity of this relationship, we performed a meta-analysis of 40 peer-reviewed, published AD transgenic mouse studies that quantitatively measured Aβ levels in brain tissue after assessing cognitive performance. We examined the relationship between Aβ levels (Aβ_40_, Aβ_42_, or the ratio of Aβ_42_ to Aβ_40_) and cognitive function as measured by escape latency times in the Morris water maze or exploratory preference percentage in the novel object recognition test. Our systematic review examined five mouse models (Tg2576, APP, PS1, 3xTg, APP(OSK)-Tg), gender, and age. The overall result revealed no statistically significant correlation between quantified Aβ levels and experimental measures of cognitive function. However, enough of the trends were of the same sign to suggest that there probably is a very weak qualitative trend visible only across many orders of magnitude. In summary, the results of the systematic review revealed that mice bred to show elevated levels of Aβ do not perform significantly worse in cognitive tests than mice that do not have elevated Aβ levels. Our results suggest two lines of inquiry: 1) Aβ is a biochemical “side effect” of the AD pathology; or 2) learning and memory deficits in AD are tied to the presence of qualitatively “high” levels of Aβ but are not quantitatively sensitive to the levels themselves.

## INTRODUCTION

Amyloid-β (Aβ) has been the most cited probable causative factor in Alzheimer’s disease (AD) since the identification of Aβ in 1984 [1]. Therefore, much research and focus within the AD community has been devoted to analyzing the relationship and possible role Aβ plays in the onset and progression of AD [2]. One of the main theories in AD causation is the Amyloid Cascade Hypothesis, which proposes that Aβ formation leads to a series of neurotoxic events that eventually lead to cell death [3]. As a result, significant amounts of research have been performed analyzing the relationship between Aβ_42_ and Aβ_40_ levels and cognitive performance in transgenic mice, and numerous articles have claimed a significant correlation between increased Aβ levels and cognitive decline (e.g., [4–8]).

One of the most common methods of experimentally assessing Aβ in AD is to subject transgenic AD mice to behavioral tests that assess memory/learning and then subsequently measure Aβ levels posttest [9]. Tg2576 is the most commonly used mouse model for this analysis as it shows elevated levels of Aβ at a young age [10]. The Morris water maze (MWM) is one of the most common memory/learning test as escape latency time in the maze is said to be correlated with spatial learning and memory [11]. Another common test is the novel object recognition (NOR) test, which tests the recognition memory of mice [12].

The ratio of Aβ_42_ to Aβ_40_ is considered just as significant, if not a better criteria for AD onset, as the ratio considers inter-individual variations in total amyloid load, whereas absolute values can have higher variance between individual test subjects [13]. Furthermore, Aβ_42_ is believed to be much more neurotoxic and thus, more connected to cell death than Aβ_40_; in fact, Aβ_40_ has even been shown to inhibit Aβ_42_ oligomerization [14]. Therefore, a higher percentage of Aβ_42_ is deemed more correlative of AD onset under the Amyloid Cascade hypothesis [14]. Together, MWM or NOR results, in combination with Aβ_42_ to Aβ_40_ ratios in tested Tg2576 mice, provide a way of analyzing the correlation between Aβ and spatial memory and learning deficiencies associated with onset and progression of AD [15].

However, recent articles have shown evidence that challenges the Amyloid Cascade Hypothesis, suggesting that Aβ is not the causative factor in AD onset (e.g., [16–20]). The goal of this study was to examine the potential relationship between experimental Aβ levels and mouse cognitive function. We perform a meta-analysis of 40 existing studies that quantitatively examined the Aβ_42_ to Aβ_40_ ratio in transgenic mouse brain tissue in relation to MWM escape latency or NOR exploratory preference.

## MATERIALS AND METHODS

We performed a systematic review of the transgenic mouse literature to examine the potential correlation between brain tissue measured Aβ levels and experimental measures of mouse cognitive function as described in detail below.

### Inclusion and Exclusion Criteria

Inclusion was based on Key term searches in PubMed to find potentially relevant publications. The terms were “Alzheimer’s Disease” (including all MESH equivalents), mouse model (e.g., Tg2576, APP, etc.; see below), and cognitive deficit measure (e.g., Morris water maze; see below). General exclusion criteria were English only and the presence of quantitative data for both Aβ_40_ and Aβ_42_ levels found in the brain. Additional, test-specific exclusion criteria are listed below for each cognitive deficit test.

### Mouse Model Descriptions

#### Tg2576

The Tg2576 mouse model expresses the human APP695 isoform with double mutation K670 N, M671 L also known as hAPPSw via the hamster prion promoter [9]. As a result this mouse exhibits levels of human amyloid-β protein precursor (Aβ PP) six times greater than that of mouse Aβ PP levels [9]. In addition the mice show higher levels of Aβ_40_ and Aβ_42_ [9]. Aβ deposits begin at 9 months of age [9].

#### APP

This model expresses hAPPSw and APP751 isoform under the control of the murine Thy1 promoter [21]. As a result this mouse exhibits levels of human Aβ PP seven times greater than that of mouse Aβ PP levels [21]. Aβ plaques begin at 6 months of age [21].

#### APP(OSK)-Tg

The APP(OSK)-Tg model expresses Aβ PP harboring the Osaka (E693Δ) mutation [22]. These mice exhibit intraneuronal Aβ oligomers and memory impairment from 8 months of age [22].

#### PS1

These mice express human presenilin with mutation M146 L or M146 V via the PDGF-β promoter [23]. This results in higher levels of endogenous mouse Aβ_1–42/43_ [23].

#### 3xTg

This triple-transgenic model of AD exhibits both Aβ and tau pathology, and mimics human AD [24].

### Cognitive Deficit Test Descriptions

#### Morris Water Maze

The MWM tests the spatial reference memory of mice [25]. The mice are usually trained to search for a hidden platform using visual cues surrounding the maze [25]. After the final day of training and usually a 24-hour waiting period, the mice are inserted once again into the maze and the time taken to find the hidden platform is measured [25]. Test-specific exclusion criteria were visible platform (i.e., use of hidden platform was a requirement), allowing the mouse to stay within the maze for more than 60 seconds, and lack of escape latency time assessment.

#### Novel Object Recognition

NOR tests the recognition memory of mice [12]. The mice are shown two objects and allowed to explore the objects [12]. One of the original two objects is then replaced with a novel object [12]. Usually after 4 hours and 24 hours the mouse is allowed to explore the new object set up [12]. The time the mice spend with the old and the new object is recorded and if the mouse spent more time exploring the novel object then it recognizes the object as different from the original set up [12]. Thus the exclusion criterion was using an assessment metric other than exploratory preference percentage.

### Analysis

Some papers presented averaged assessments while others presented all data from multiple trials. Consequently, we averaged within-paper multi-trial data so that all papers would be given equivalent weighting in the review.

The data were divided into the following mouse age groups: 6–11 months, 12–14 months, and 15–20 months. Separately, the data was broken down into groups by gender.

Prior to conducting a statistical analysis of significance, the distribution of the data was first determined. Through the analysis of normal probability plots of the data, it was found that the data fell into a normal distribution. Statistical significance was subsequently assessed with an F-test of the least-squares regression line of cognitive deficit test metric versus log of Aβ levels or log of Aβ_42_/Aβ_40_ ratios. Note that the log of Aβ level was utilized due to the large variation of Aβ level magnitudes among the pooled studies. Despite the large number of examined correlations, a very generous *p*-value < 0.05 was used as the threshold for potential significance based on the ultimate findings.

## RESULTS

A total of 40 peer-reviewed, published scientific articles met the study inclusion criteria. From these articles, a total of 230 data points were extracted to assess the relationship between Aβ level and AD transgenic mouse cognitive performance. The data was categorized and analyzed by mouse type, Aβ type, Aβ solubility, gender, age, and cognitive test utilized (see Table 1). Raw soluble and insoluble Aβ levels (Aβ_40_ and Aβ_42_) as well as Aβ_42_ to Aβ_40_ ratios were examined for each category shown.

**Fig. 1:**
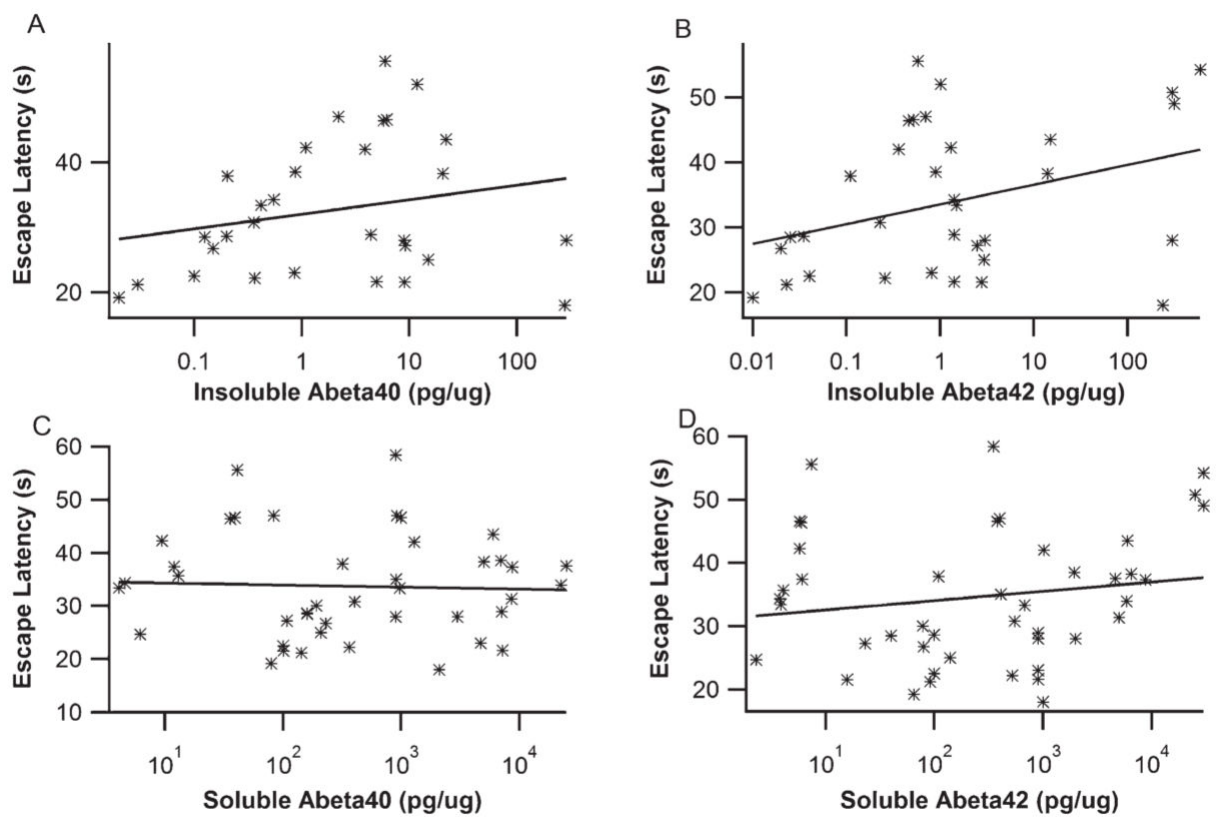
The log of raw Aβ_40_ and Aβ_42_ soluble and insoluble levels per area of tissue in the brain is plotted versus Morris water maze escape latency in Tg2576 mice. Only the insoluble level of Aβ_42_ was found to be significantly correlated with Morris water maze escape latency. No correlation was found between escape latency and insoluble Aβ_40_, soluble Aβ_40_, or soluble Aβ_42_. A) Insoluble levels of Aβ_40_, *r*^2^ = 0.049, *p*-value = 0.24. B) Insoluble levels of Aβ_42_, *r*^2^ = 0.125, *p*-value = 0.0043. C) Soluble levels of Aβ_40_, *r*^2^ = 0.0017, *p*-value = 0.80. D) Soluble levels of Aβ42, *r*^2^ = 0.027, *p*-value = 0.281.

We began by examining the data for the raw Aβ_42_ and Aβ_40_ levels. MWM escape latency was plotted versus both the log of soluble and insoluble Aβ_42_ and Aβ_40_ levels for all of the Tg2576 transgenic mouse studies. As shown in Fig. 1, raw Aβ levels vary greatly in magnitude among the data extracted from 21 different articles. An assessment of the relationship of MWM escape latency versus log of insoluble levels of Aβ_40_ result in a *r*^2^ value of 0.049 and a *p*-value of 0.24, and the log of insoluble levels of Aβ_42_ result in a *r*^2^ value of 0.125 and a *p*-value of 0.043. Similarly, the log of soluble levels of Aβ_40_ result in a *r*^2^ value of 0.0017 and a *p*-value of 0.80, while the log of soluble levels of Aβ_42_ result in a *r*^2^ value of 0.027 and a *p*-value of 0.281. In summary, these statistical results reveal that only insoluble Aβ_42_ concentration was potentially significant. Note, however, three of the four plots show the same trend toward increasing escape latency across four or five orders of magnitude of concentration.

**Fig. 2:**
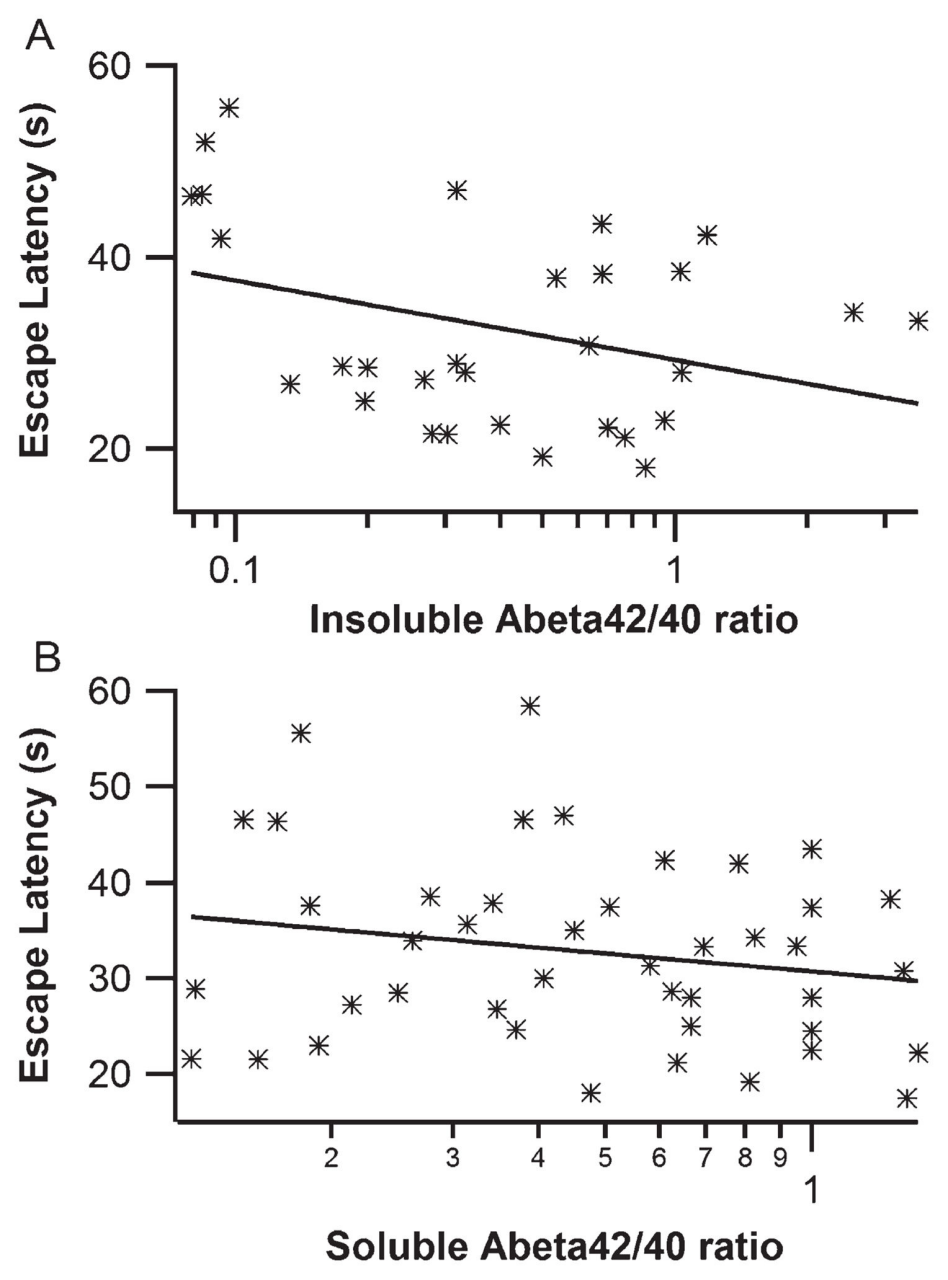
Extracted and pooled Tg2576 data from 21 studies showed no statistical correlation between Morris water maze escape latency and the log of Aβ_42_ to Aβ_40_ ratio in the brain tissue of transgenic mice. A) soluble ratio between levels of Aβ_42_ to Aβ_40_, *r*^2^ = 0.039, *p*-value = 0.206. B) insoluble ratio, *r*^2^ = 0.118, *p*-value = 0.0625.

Next, we examined the MWM escape latency versus the Aβ_42_/Aβ_40_ ratio, since the ratio is thought to be a better assessment measure than the raw Aβ levels [13]. Figure 2 shows the overall results of the meta-analysis for both the soluble and insoluble data points in Tg2576 mice. The effect of the log of insoluble Aβ ratio on average escape latency in the MWM was found to have an *r*^2^ value of 0.118 and a *p*-value of 0.0625, showing that there was no correlation between soluble Aβ_42_ to Aβ_40_ ratio and escape latency results. Results for log of soluble Aβ ratio were similarly non-correlative with an *r*^2^ value of 0.039 and a *p*-value of 0.206. However both soluble and insoluble weakly trend toward higher ratios corresponding to lower escape latencies.

**Fig. 3:**
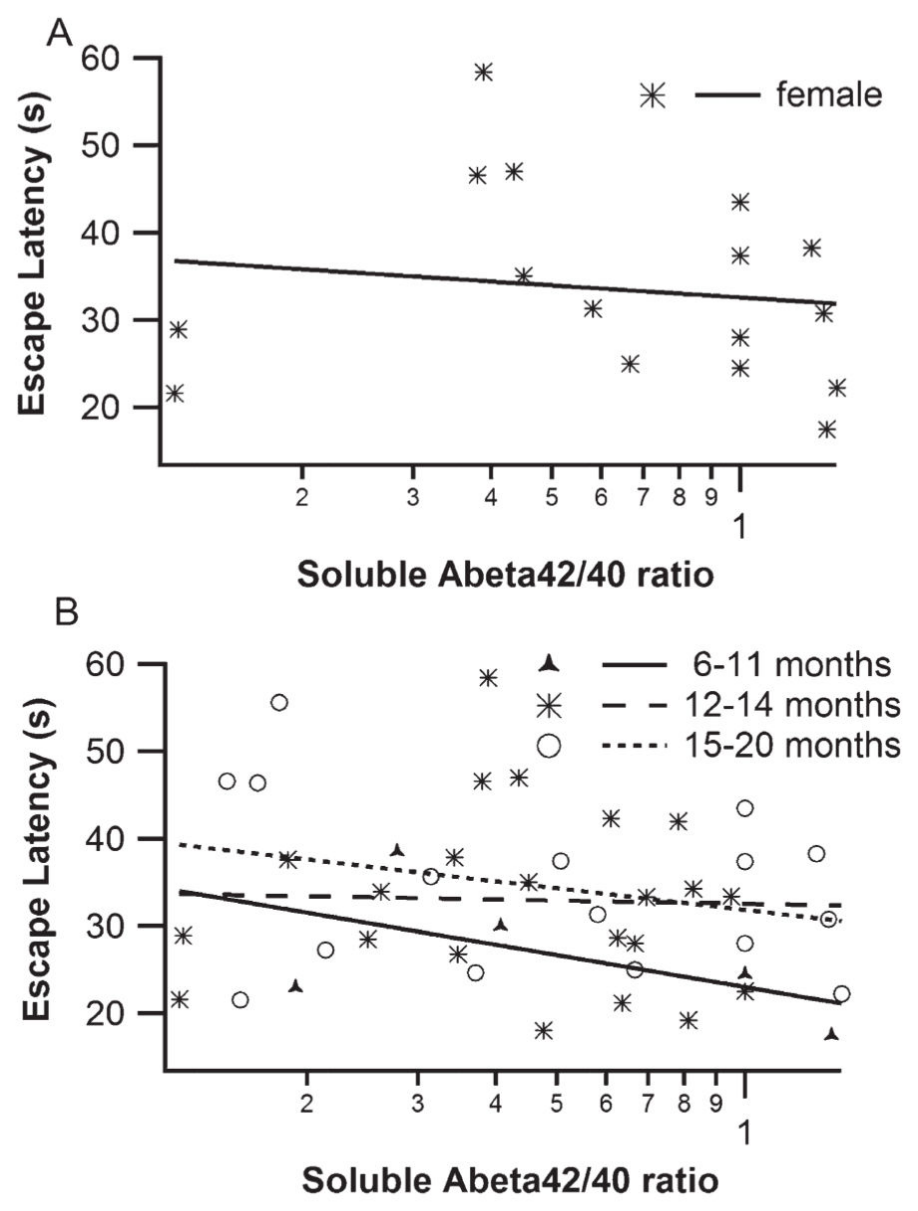
Age and Gender Separation data showed no statistical correlation between Morris water maze escape latency and the log of Aβ_42_ to Aβ_40_ ratio in the brain tissue of transgenic mice. Extracted data for the Tg2576 mice were separated into groups based on age and gender differences used in the included studies. A) All female mice, *r*^2^ = 0.02, *p*-value = 0.6. B) Mice ages 6–11 months, *r*^2^ = 0.31, *p*-value = 0.33. Mice ages 12–14 months, *r*^2^ = 0.001, *p*-value = 0.88. Mice ages 15–20 months, *r*^2^ = 0.093, *p*-value = 0.25.

To assess the possible effects of gender and age, we split the full Tg2576 data set into different corresponding groups. Figure 3 shows MWM escape latency versus the log of Aβ_42_/Aβ_40_ ratio for Tg2576 separated by age and gender of the mice. The 6–11 month group has an *r*^2^ value of 0.31 and a *p*-value of 0.33; the 12–14 month group has an *r*^2^ value of 0.001 and *p*-value of 0.88, and the 15–20 month group has an *r*^2^ value of 0.093 and a *p*-value of 0.25. These results again fail to reach our threshold for statistical correlation between MWM escape latency and the Aβ_42_/Aβ_40_ ratio. The results for the female-only Tg2576 groups are similarly non-correlative. The female data set has an *r*^2^ value of 0.02 and a *p*-value of 0.6. There were an insufficient number of male-only studies to perform a correlation analysis.

**Fig. 4:**
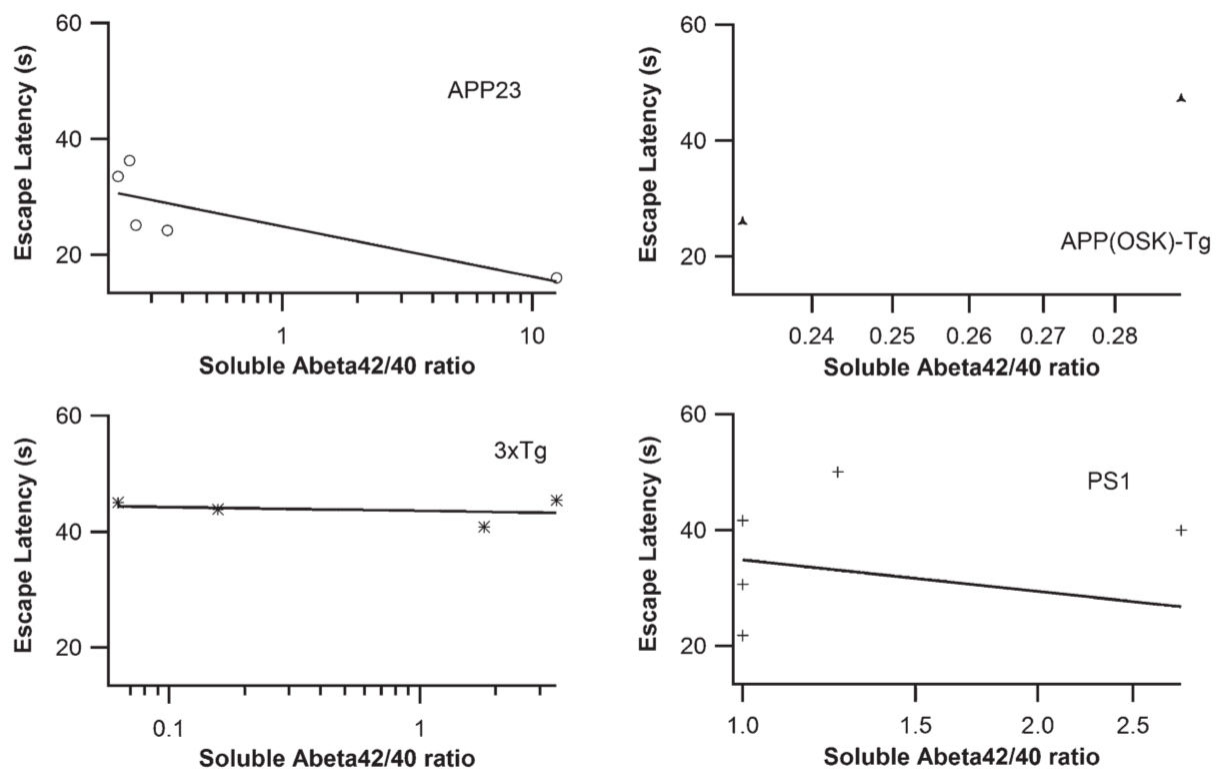
APP, APP(OSK)-Tg, 3xTg, and PS1 mice showed no correlation between Morris water maze escape latency and the log of Aβ_42_ to Aβ_40_ ratio in brain tissue. Extracted data for A) APP mice, *r*^2^ = 0.65, *p*-value = 0.098. B) APP(OSK)-Tg mice, too few points. C) 3xTg mice, *r*^2^ = 0.072, *p*-value = 0.73. D) PS1 mice, *r*^2^ = 0.10, *p*-value = 0.60.

Finding no correlation between Aβ_42_/Aβ_40_ ratio and MWM latency in the Tg2576 mouse studies, we subsequently examined other mouse models for a possible correlation, including the APP(OSK)-Tg, PS1, 3xTg, and APP. However, as shown in Fig. 4, there is no statistically significant correlation between MWM escapelatency and Aβ_42_/Aβ_40_ ratio for any of these four additional mouse models. With only two APP(OSK)-Tg data points, the correlation could not be assessed. The PS1 mice have an *r*^2^ value of 0.10 and *p*-value of 0.60. The 3xTg mice have an *r*^2^ value of 0.072 and *p*-value of 0.73. Finally, the APP mice have an *r*^2^ value of 0.65 and *p*-value of 0.098. Therefore, the specificity of mouse model type does not appear to explain the apparent absence of correlation between quantitative Aβ_42_/Aβ_40_ ratios and MWM escape latency.

**Fig. 5:**
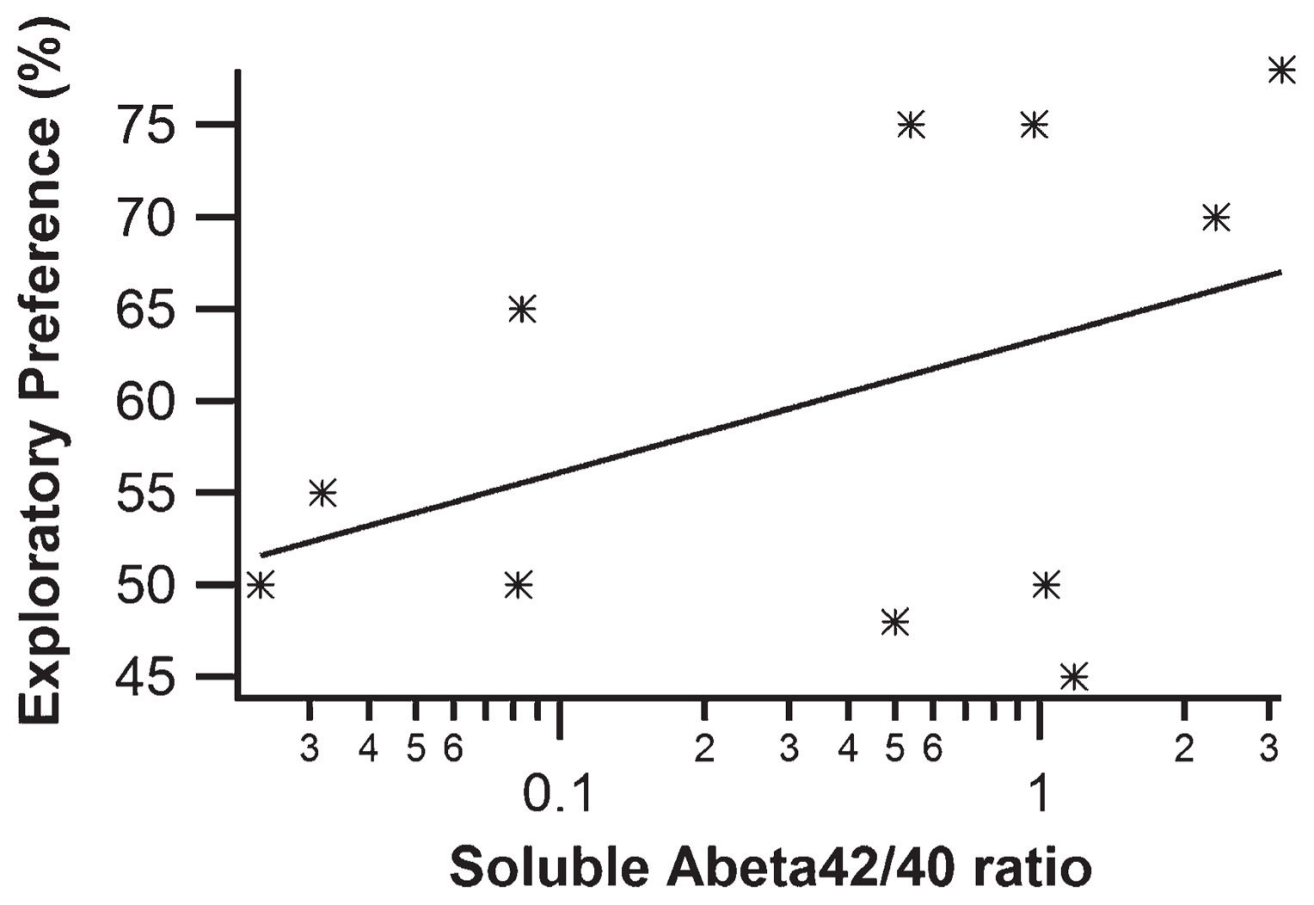
Extracted data for Tg2576 mice subjected to the novel object recognition test showed no correlation between exploratory preference percentage and the log Aβ_42_ to Aβ_40_ ratio in brain tissue, *r*^2^ = 0.19, *p*-value = 0.19.

Finding no consistent correlation between Aβ_42_/Aβ_40_ ratio and MWM escape latency in our systematic review of the five examined transgenic mouse models, we subsequently examined a different measure of cognitive function in transgenic AD mice—exploratory preference percentage in the NOR test. Data for the NOR test was pooled and systematically reviewed from 8 Tg2576 mouse articles. However, our statistical assessment of the combined NOR set of 19 data points reveals no statistically significant correlation between exploratory preference percentage and Aβ_42_ to Aβ_40_ ratio, as the *r*^2^ value was 0.079 and the *p*-value was 0.24 (Fig. 5).

## DISCUSSION

The results for our systematic review of 40 different transgenic AD mouse research articles showed no reliable correlation between Aβ levels (Aβ_40_, Aβ_42_, and Aβ_42_/Aβ_40_) and experimental assessments of cognitive function (maze escape latency or exploratory preference percentage). Despite examining results from two different cognitive tests (MWM and NOR), five different mouse models (Tg2576, APP, PS1, 3xTg, and APP(OSK)-Tg) and considering potential gender and age differences, no statistical relationship could be identified linking Aβ to mouse cognitive decline in AD. However, enough of the trends were of the same sign to suggest that there probably is a very weak qualitative trend visible only across many orders of magnitude. In summary, the results of the systematic review revealed that mice bred to show elevated levels of Aβ do not perform significantly worse in cognitive tests than mice that do not have elevated Aβ levels.

It is possible that a particular mouse model or experimental cognitive function assessment combination *not* examined as part of this systematic review may reveal a correlation between Aβ and AD cognitive decline. However, given the size and statistical power of the data sets examined, we propose two more likely hypotheses to explain the lack of correlation between elevated quantitative Aβ levels and poor cognitive performance: 1) Learning and memory deficits in AD are tied to the presence of qualitatively “high” levels of Aβ but are not quantitatively sensitive to the numerical values of the levels, themselves; or 2) It is possible that Aβ, itself, is not directly responsible for AD-related cognitive impairment, but rather is simply a “side effect” of the ongoing biochemical and cellular processes involved in the AD pathology.

In fact, more recent research has cited evidence against the Amyloid Cascade Hypothesis. One of the most cited points against the Amyloid Cascade Hypothesis is that, after more than two decades of research, not a single viable treatment for AD has resulted from treatments based on this hypothesis [20, 26–28]. Another point made against the Amyloid hypothesis is that while Aβ levels are often elevated among AD patients [29], the levels, themselves, are not consistent indicators of clinical AD advancement and progression among individuals [5, 30]. In fact, in some studies, several AD patients who had severely deteriorated memory showed no plaques postmortem [5, 26–28, 30, 31]. While transgenic mouse experimental correlations are helpful for understanding disease mechanisms, they should not be misunderstood as “clinical” correlations. Nonetheless, despite the fact that the sporadic forms of clinical AD do not share the same underlying genetics of AD mice, an examination of quantitative Aβ levels in comparison to cognitive function appears to be the similar; that is, in both human AD patients and transgenic mouse models, there is a lack of a direct, quantitative correlation between Aβ levels and cognitive performance.

Furthermore, it has been proposed that decline in brain metabolic activity, which is tightly linked to synaptic activity, actually underlies both the cognitive decline in AD and the deposition of Aβ [23]. The fact that vast overproduction of Aβ peptides in the brain of transgenic mouse models fails to cause overt neurodegeneration raises the question as to whether accumulation of Aβ peptides is indeed the primary culprit for neurodegeneration in AD [24]. There is increasing evidence to suggest that Aβ/amyloid-independent factors, including the actions of AD-related genes (microtubule-associated protein tau, polymorphisms of apolipoprotein E4), inflammation, and oxidative stress, also contribute to AD pathogenesis [32, 33]. Thus, while no one argues that Aβ accumulation is present in both clinical AD and AD transgenic mice, Aβ accumulation, itself, may not directly result in associated cognitive decline. The results of this large systematic review of AD transgenic mouse data, in conjunction with the aforementioned research, support the contention that the debate over the possible role of Aβ in AD cognitive decline needs to be re-ignited and fueled by research into other possible hypotheses and explanations.

## Figures and Tables

**Table 1:** Transgenic mouse studies included in the systematic review

Model	Type	Included articles	Soluble data points	Insoluble data points	Total data points	Reference
Morris water maze						
Tg2576	All	21	86	63	149	[34–55]
	Female	8	28	20	48	[35, 41, 43, 45, 48–51]
	Male	1	4	4	8	[40]
	6–11 Mo.	4	9	6	15	[34–37]
	12–14 Mo.	10	47	35	82	[38–47]
	15–20 Mo.	8	32	22	54	[48–55]
App	All	3	10	8	18	[56–58]
PS1	All	5	8	14	22	[59–62]
APP(OSK)	All	1	4	4	8	[22]
3xTg	All	2	8	6	14	[63, 64]
Novel object recognition						
Tg2576	All	8	11	8	19	[65–72]
